# Psychological distress among family caregivers of persons with Alzheimer’s disease and related dementias in Uganda

**DOI:** 10.1186/s12877-024-05190-z

**Published:** 2024-07-15

**Authors:** Joy Louise Gumikiriza-Onoria, Janet Nakigudde, Roy William Mayega, Bruno Giordani, Martha Sajatovic, Mark Kaddu Mukasa, Dennis Buwembo, Kamada Lwere, Noeline Nakasujja

**Affiliations:** 1 Department of Psychiatry, School of Medicine, Makerere University College of Health Sciences, Kampala, Uganda; 2 Department of Psychiatry, Makerere University College of Health Sciences, School of public Health, Kampala, Kampala, Uganda; 3 Department of Psychiatry, Makerere University College of Health Sciences, School of Biomedical Sciences, Kampala, Uganda; 4 Department of Psychiatry; University of Michigan Faculty Ombuds; Associate Director, Michigan Alzheimer’s Disease Center; Senior Director of the Mary A. Rackham Institute (MARI), Ann Arbor, MI USA; 5 Neurological and Behavioral Outcomes Center, University Hospitals Cleveland Medical Center, Case Western Reserve University School of Medicine, Cleveland, OH USA

**Keywords:** Alzheimer’s disease, Related dementias (ADRD), Caregiver stress, Psychological distress, Quality of life (QoL), Depression, Uganda, Familial caregiving

## Abstract

**Background:**

Alzheimer's disease and related dementias (ADRD) present growing global health challenges, especially in aging populations, such as Uganda. In Uganda, familial caregiving, predominantly undertaken by female relatives, is the primary form of support provided to patients with ADRD. Cultural stigma around dementia and limited access to support services amplify caregivers' challenges. This study examined psychological distress, depression, and quality of life (QoL) among family caregivers of patients with ADRD in Wakiso District, Uganda.

**Methods:**

This cross-sectional study involved 90 caregivers from three sub-counties in Wakiso, selected through purposive sampling to capture diverse experiences. Participants included caregivers aged 18 years and older who were knowledgeable and had cared for a person with ADRD for not less than six months, with those providing more than 70% of physical care being prioritised. Data were collected using the Kessler Psychological Distress Scale, the Caregiver Dementia Quality of Life Measurement Scale, and the Center for Epidemiologic Studies Depression Scale, with an 80% response rate achieved through local collaboration. The statistical analyses focused on psychological distress, QoL, and depression.

**Results:**

The study included 82.2% females and 17.8% males, with a median age of 52 years for females and 35 years, respectively. Females were more likely to be single or widowed, whereas males were more likely to be married. The study revealed a high prevalence of psychological distress and depression among caregivers (64.4%) regardless of sex. The analysis indicated that having children was a significant predictor of better QoL (OR 3.04, 95% CI 1.79–5.66, *p* = 0.034) and a lower risk of depression (OR 0.10, 95% CI 0.01–0.86, *p* = 0.036). No other sociodemographic factors were significantly associated with health outcomes across the models.

**Conclusion:**

Our findings revealed a heavy burden of psychological distress and depression among Ugandan caregivers of patients with ADRD, highlighting the need for structured support systems, including mental health services and gender-responsive interventions in low-resource settings.

## Background

Alzheimer's disease and related dementia (ADRD) are significant and growing global health challenges. The prevalence of ADRD is increasing with the aging population worldwide, placing strain on healthcare systems, families, and caregivers [1–4]. Given the growing significance of ADRD and the pivotal role that caregivers play, understanding the specific challenges and distress they face in this context is crucial.

In Uganda, as in many other African countries, familial caregiving remains the primary means of support for individuals with ADRD [5]. Familial caregivers play an indispensable role in providing emotional, physical, and logistical support. Traditional family structures in Uganda often place responsibility on caring for family members, primarily female relatives, who may have limited formal education and resources to manage the complex needs of individuals with ADRD [6]. However, this essential service often comes at considerable personal cost. Caregivers of individuals with ADRD often experience high levels of stress, emotional exhaustion, and psychological distress [7–9]. These experiences can be exacerbated in settings with limited access to formal health and social support services, as is the case in many parts of sub-Saharan Africa, including Uganda [6, 10].

Cultural beliefs and stigma associated with dementia in Africa can further compound caregiver distress [11, 12]. Misunderstandings about the nature and causes of dementia can lead to delays in seeking medical advice, isolation, and discrimination [13]. Moreover, in settings where resources are constrained, caregivers may face additional challenges such as limited access to accurate information about the disease, lack of appropriate medical care, and financial strain [14]. In this study, we aimed to systematically investigate psychological distress and its associated factors, such as depression and Quality of Life (QoL), in family caregivers of patients with ADRD in Uganda.

## Methods

This study was conducted in Wakiso District, which is located in the Central Region of Uganda. Recent estimates suggest that the population of the district is over two million, with a notable proportion of individuals aged 65 years and above [15]. In Uganda, older adults often rely on their social networks for care and economic support, a trend that is well-documented along with the complexities and limitations of these support systems [16]. Our research was conducted in three representative sub-counties of the district: the urban area of Nansana and the rural areas of Busukuma.

### Participant selection

This study utilized a purposive sampling method to select 90 caregivers from a pre-existing cohort of 500 individuals initially formed for a community assessment study on Alzheimer's Disease and Related Dementias (ADRD) in the Nansana and Busukuma sub-counties of Uganda's Wakiso District. These areas were chosen because of their diverse urban and rural populations, which offer a broad perspective on caregiving dynamics. The cohort comprised caregivers of patients with ADRD diagnosed by psychiatrists and psychologists using comprehensive diagnostic criteria, including neurocognitive evaluations and CT scans, ensuring the accuracy of patient conditions.

Participants included caregivers aged 18 years and older who were knowledgeable and had cared for a person with ADRD for not less than six months, with those providing more than 70% of physical care being prioritized. The sampling aimed to capture a wide range of first-hand caregiving experiences, targeting specific demographic criteria such as age, sex, and active caregiver status to ensure comprehensive data collection. We collaborated with Village Health Teams (VHTs) and local council leaders to identify households with diagnosed ADRD patients, aligning with previous studies that assessed caregiver stress within these communities.

Cochran’s formula [17] was used to estimate the sample size for a cross-sectional study, which determined that a sample size of 90 caregivers would be sufficient to ensure adequate coverage and account for potential non-responses or dropouts. However, to maintain the integrity of our study, we excluded caregivers who were critically ill, not primary caregivers, or under 18 years of age. This careful selection process led to approaching 113 homesteads, achieving an 80% caregiver response rate, which underscores the method’s effectiveness and the alignment of our research.

### Data collection procedures

A team of three experienced research assistants with backgrounds in community psychology, social work, and nursing collected data. The interviews lasted for 90–120 min. The interviews were conducted in either English or Luganda, the dominant language used in the region, to ensure clear communication. To ensure that the study met ethical standards, clearance was obtained from the Makerere School of Medicine Research and Ethics Committee and Uganda National Council for Science and Technology (HS2909ES) and registered at the Pan African Clinical Trials Registry (PACTR202211700581839). Before data collection, each potential participant was approached individually to explain the purpose, nature, potential benefits, and risks of the study. Participants were informed that their participation was voluntary and that they had the right to withdraw without consequences. Written informed consent was obtained from those willing to participate after ensuring their understanding and comfort. Data were stored securely, ensuring limited access to authorized personnel and maintaining the anonymity and privacy of the participants.

### Data collection instruments

Sociodemographic details, such as age, sex, educational background, occupation, comorbidities, and marital status, were collected using a brief questionnaire. Psychological distress was assessed using the Kessler Psychological Distress Scale (K10) [18, 19], which has an internal consistency of 0.78. The K10-scale was employed alongside the Centre for Epidemiologic Studies Depression Scale (CES-D) [20], which has an internal consistency of 0.79, to provide a comprehensive evaluation of psychological states. The use of both scales allows for a broader assessment of psychological distress and specific depressive symptoms, ensuring a robust measurement of mental health outcomes among caregivers. The Caregiver Dementia Quality of Life Measurement (C-DMEQOL) was used to evaluate caregiver quality of life, featuring an internal consistency of 0.87 and including five key domains: emotional well-being, physical health, social interaction, financial well-being, and caregiving satisfaction. This combination of instruments facilitated a detailed analysis of both the psychological and situational aspects of caregivers' experiences.

### Data management and statistical analysis

A formal analysis of sociodemographic attributes was conducted using frequency distributions, and gender-based differences were examined using a chi-square test. The Mann–Whitney U test was used to investigate age-related disparities by sex. Our study aimed to assess the degree of psychological distress among caregivers of individuals with ADRD, with the primary independent variables encompassing potential distress triggers related to caregivers’ demographics. The reliability of the K10-scale, C-DMEQOL, and CES-D was confirmed using Cronbach's alpha. The outcome variables of interest included the prevalence of psychological distress, quality of life, and depression indicators determined using proportions. A cut-off score of 22 on this scale indicates severe psychological distress. A score of 95 or higher on the C-DMEQOL indicated a good quality of life, and scores of 16 or higher on the CES-D indicated the presence of depression. Multivariate logistic regression analyses were performed to explore the relationships between various factors and outcomes, calculating both crude and adjusted odds ratios (OR), along with 95% confidence intervals (CI), to identify the strength and orientation of these associations. Statistical significance was set at p < 0.05, and all analytical procedures were performed using the Stata 17.

## Results

### Overall sample description

This study included 90 participants, predominantly females (82.2%), while males constituted 17.8% of the sample. Females had a median age of 52 years, and males had a median age of 35 years, with age differences approaching statistical significance (*p* = 0.052). Regarding marital status, females were more likely to be single (34.2%) or widowed (13.7%), in contrast to males who were predominantly married (56.3%). Educational attainment varied significantly between genders; 52.7% of females had primary or lower education compared to 25.0% of males. The majority of female (87.8%) and male (75.0%) participants had children. Reported comorbidities were high among participants, with distinct patterns observed between sexes: 62.5% of males reported having HIV, significantly higher than females, and 59.5% of females reported urinary tract infections (UTIs) with a significant difference noted (*p* = 0.040). Further details are presented in Table 1.

**Table 1:** Socio-Demographics Characteristics. This table presents the socio-demographic characteristics of the study participants, categorized by gender. The factors assessed include age, marital status, education level, presence of children, self-reported comorbidities (HIV and UTI), source of income in UGX, and monthly income. Statistical comparisons between male and female participants were conducted using *p-*values to determine the significance of observed differences

Factor	Gender		*p-*value
	Male (*n=*16; 17.8%)	Female (*n=*74; 82.2%)	
Age (years)	35 (23,43)	52 (37,61)	0.052
Marital status			
Widow	0 (0.0%)	10 (13.7%)	0.231
Married	9 (56.3%)	25 (34.2%)	
Divorced	1 (6.3%)	3 (4.1%)	
Single	6 (37.5%)	35 (48.0%)	
Education level			
Primary & below	4 (25.0%)	39 (52.7%)	0.115
Secondary	10 (62.5%)	31 (41.9%)	
Tertiary	2 (12.5%)	4 (5.4%)	
Children			
No	4 (25.0%)	9 (12.2%)	0.185
Yes	12 (75.0%)	65 (87.8%)	
Comorbidity			
HIV	10 (62.5%)	29 (39.2%)	0.088
UTI	5 (31.3%)	44 (59.5%)	0.040
Source of income			
None	8 (50.0%)	35 (47.3%)	0.119
Farming	1 (6.3%)	15 (20.3%)	
Relatives	0 (0.0%)	10 (13.5%)	
Business	6 (37.5%)	11 (14.9%)	
Professional job	1 (6.3%)	3 (4.1%	
Monthly income			
100,000 & below	10 (62.5%)	61 (82.4%)	0.076
Over 100,000	6 (37.5%)	13 (17.6%)	

### Scale scores and prevalence

Table 2 displays the average QoL, psychological distress, and depression scores according to sex. No significant disparities in these scores were detected between males and females. The prevalence of psychological distress was 64.4%, and that of QoL revealed a poor QoL (52.2%). Furthermore, 64.4% of the individuals exhibited symptoms of depression.

**Table 2:** Scale scores and prevalences. This table presents the frequency and percentage distribution of quality of life (QOL), psychological distress, and depression among the study participants, categorized by gender. It provides a comparison of these health outcomes between male and female caregivers of individuals with ADRD

Factor	FREQUENCY (PERCENTAGE)	Gender	
		Male (*n=*16)	Female (*n=*74)
QOL			
Poor	47 (52.2%)	8 (50%)	39 (52.7%)
Good	43 (47.8%)	8 (50%)	35 (47.3%)
Psychological distress			
No/mild distress	32 (35.6%)	4 (25.0%)	28 (37.8%)
Moderate/severe distress	58 (64.4%)	12 (75.0%)	46 (62.2%)
Depression			
No Depression	32(35.6%)	3 (18.75%)	12 (16.2%)
Symptomatic Depression	58 (64.4%)	13 (81.25%)	62 (83.8%)

### Multivariate linear regression models

Tables 3, 4 and 5 present the results of the multivariate linear regression analysis of QoL, Psychological Distress, and Depression. The analysis revealed that having children was a significant predictor of better QOL in the adjusted model (OR 3.04, 95% CI 1.79–5.66, *p* = 0.034). Psychological distress was significantly associated with poorer QOL in the adjusted model for distress outcomes (OR 3.25, 95% CI 1.01–10.82, *p* = 0.044), and having children predicted lower risk of depression (OR 0.10, 95% CI 0.01–0.86, *p* = 0.036) in the adjusted depression model. No other socio-demographic factors showed significant associations with health outcomes across the models.

**Table 3:** Results for fitting a multivariate logistic regression model. (Outcome: QOL). This table presents the crude and adjusted odds ratios (OR) with 95% confidence intervals (CI) and *p-*values for the association between various sociodemographic factors and health outcomes, including psychological distress and depression, among caregivers. The factors analyzed include age, gender, marital status, education level, presence of children, comorbidities, source of income, monthly income, psychological distress, and depression

Factor	level	Crude estimates		Adjusted model	
		Odds ratio (95% CI)	*p-*value	Odds ratio (95% CI)	*p-*value
Age	Mean (SD)	0.99 (0.97,1.01)	0.354	0.96 (0.93,1.00)	0.076
Gender	Male	Reference			
	Female	0.90 (0.30,2.65)	0.844	0.55 (0.10,2.90)	0.481
Marital status	Widow	Reference			
	Married	1.27 (0.31,5.20)	0.588	1.14 (0.20,6.51)	0.523
	Divorced	-		-	
	single	0.78 (0.20,3.13)		0.57 (0.09,3.41)	
Education level	Primary & below	Reference			
	Secondary	0.82 (0.35,1.94)	0.699	1.05 (0.30,3.67)	0.544
	Tertiary	0.48 (0.08,2.89)		0.25 (0.02,3.29)	
Children	No	Reference			
	Yes	2.56 (1.47,5.19)	0.045	3.04 (1.79,5.66)	0.034
Comorbidity	HIV	1.54 (0.66,3.56)	0.315	1.94 (0.61,4.56)	0.315
	UTI	0.78 (0.34,1.78)	0.550	0.58 (0.34,2.78)	0.550
Source of income	None	Reference	0.552		
	Farming	1.35 (0.42,4.27)		1.51 (0.37,6.23)	0.536
	Relatives	0.45 (0.10,1.97)		0.44 (0.08,2.56)	
	Business	0.73 (0.24,2.28)		0.63 (0.11,3.55)	
	Professional job	3.14 (0.30,32.65)		5.85 (0.18,187.42)	
Monthly income	100,000 & below	Reference			
	Over 100,000	0.42 (0.14,1.24)	0.117	0.14 (0.02,1.01)	0.051
Psychological distress	No/mild distress	Reference			
	Moderate/severe distress	1.91 (0.79,4.62)	0.149	3.14 (0.96,10.29)	0.058
Depression	No Depression	Reference			
	Depression	2.74 (0.68,11.07)	0.158	2.39 (0.48,11.96)	0.288

**Table 4:** Results for fitting a multivariate logistic regression models. (Outcome: Psychological distress. This table presents the crude and adjusted odds ratios (OR) with 95% confidence intervals (CI) and *p-*values for the association between various sociodemographic factors and health outcomes, including quality of life (QOL) and depression, among caregivers. The factors analyzed include age, gender, marital status, education level, presence of children, comorbidities, source of income, monthly income, QOL, and depression

Factor	level	Crude estimates		Adjusted model	
		Odds ratio (95% CI)	*p-*value	Odds ratio (95% CI)	*p-*value
Age	Mean (SD)	1.00 (0.98,1.03)	0.731	1.01 (0.97,1.05)	0.625
Gender	Male	Reference			
	Female	0.55 (0.16,1.86)	0.335	0.67 (0.144,3.09)	0.606
Marital status	Widow	Reference			
	Married	0.6 (0.11,3.34)	0.375	0.50 (0.08,3.25)	0.469
	Divorced	0.25 (0.02,3.04)		1.12 (0.05,22.93)	
	single	0.32 (0.06,1.69)		0.30 (0.05,1.97)	
Education level	Primary & below	Reference			
	Secondary	0.75 (0.31,1.85)	0.820	0.53 (0.16,1.81)	0.510
	Tertiary	0.97 (0.16,5.92)		1.29 (0.11,15.08)	
Children	No	Reference			
	Yes	0.28 (0.06,1.38)	0.118	0.09 (0.01,0.67)	0.019
Comorbidity	HIV	0.66 (0.28,1.57)	0.344	-	-
	UTI	1.32 (0.56,3.14)	0.530	-	-
Source of income	None	Reference			
	Farming	0.38 (0.12,1.22)	0.367	0.37 (0.91,1.53)	0.514
	Relatives	0.72 (0.18,2.99)		0.92 (0.15,5.64)	
	Business	1.57 (0.43,5.70)		1.97 (0.30,13.07)	
	Professional job	1.45 (0.14,15.21)		2.50 (0.14,45.60)	
Monthly income	100,000 & below	Reference			
	Over 100,000	1.72 (0.56,5.31)	0.347	1.43 (0.24,8.47)	0.695
QOL	Good	Reference			
	Poor	1.91 (0.79,4.62)	0.149	3.25 (1.01,10.82)	0.044
Depression	No depression	Reference			
	depression	1.60 (0.45,5.74)	0.467	1.05 (0.22,4.94)	0.947

**Table 5:** Results for fitting a multivariate logistic regression model. (outcome: Depression). This table presents the crude and adjusted odds ratios (OR) with 95% confidence intervals (CI) and *p-*values for the association between various sociodemographic factors and health outcomes, including quality of life (QOL) and psychological distress, among caregivers. The factors analyzed include age, gender, marital status, education level, presence of children, comorbidities, source of income, and monthly income

Factor	Level	Crude estimates		Adjusted model	
		Odds ratio (95% CI)	***p-***value	Odds ratio (95% CI)	***p-***value
Age	Per unit increase	0.99 (0.96,1.01)	0.317	0.98 (0.94,1.02)	0.335
Gender	Male	**Reference**			
	Female	0.79 (0.25,2.52)	0.692	1.26(0.28,5.75)	0.762
Marital status	Widow	**Reference**			
	Married	2.17(0.49,9.64)	0.750	2.56(0.45,14.42)	0.185
	Divorced	0.67(0.06,6.87)		**2.53(0.14,46.12)**	
	single	0.85(0.21, 3.48)		0.60(0.11,3.38)	
Education level	Primary & below	**Reference**			
	Secondary	1.03(0.42,2.54)	0.811	0.56(0.16,1.95)	0.179
	Tertiary	0.54(0.09,3.00)		0.05(0.01,1.22)	
Children	No	**Reference**			
	Yes	0.28(0.06,1.37)	0.118	0.10(0.01,0.86)	0.036
Comorbidity	HIV	0.97(0.41,2.33)	0.953	-	-
	UTI	0.89(0.37,2.13)	0.798	-	-
Source of income	None	**Reference**			
	Farming	1.74(0.51,5.88)	0.481	4.48(0.91,22.0)	0.170
	Relatives	1.19(0.29,4.82)		1.10(0.20,6.21)	
	Business	2.57(0.72,9.18)		4.01(0.63,25.45)	
	Professional job	**-**		-	
Monthly income	100,000 & below	**Reference**			
	Over 100,000	1.72(0.56,5.31)	0.347	0.63(0.10,3.96)	0.621
Quality of life	Good	**Reference**			
	Poor	1.29(0.54,3.06)	0.170	3.45 (0.64,8.70)	0.015
Psychological distress	No/mild distress	**Reference**			
	Moderate /severe disorder	2.13(1.13,5.21)	0.038	2.06 (1.21,5.28)	0.021

## Discussion

This cross-sectional study aimed to investigate psychological distress and associated factors, such as depression and Quality of Life (QoL), among family caregivers of patients with ADRD in Uganda. We conducted a study on 90 caregivers of patients with ADRD in Wakiso, Uganda, to provide invaluable information regarding their demographic profiles and psychological experiences. The majority of participants were female (82.2%), with a median age of 52 years for females and 35 years for males. The study also found that single female caregivers accounted for 48.0% of the group, whereas married males accounted for 56.3%. Male educational attainment was skewed towards secondary levels, whereas females had a higher prevalence of primary or lower education. The study further highlighted the socioeconomic constraints and health challenges faced by this caregiving group, including comorbidities such as HIV and UTIs, and sources of income. The study further discovered that psychological distress affected 64.4% of caregivers, and quality of life scores were nearly even, emphasizing that significant toll caregiving takes on individuals and the need for attention to both the economic and psychosocial dimensions of caregiver support.

### Prevalence of psychological distress and depression

The findings of our study, which indicate a high prevalence of psychological distress (64.4%) and depression (64.4%) among caregivers, are consistent with the existing literature on the impact of caregiving on mental health [7]. The results of our research highlight a crucial and often-overlooked aspect of the ADRD caregiving experience, particularly in low- and middle-income settings such as Uganda. The significant proportion of caregivers who reported symptoms of psychological distress and depression (64.4%) highlighted a substantial mental health challenge within the Wakiso community. This prevalence is consistent with previous research in different settings, underscoring the significant psychological toll that caregiving can have on individuals [7].

Furthermore, the high comorbidity of psychological distress and depression among caregivers highlights the multifaceted nature of their burden. Caregivers not only face the emotional strain of witnessing the progressive decline of their loved ones but are also subject to the tangible hardships of providing care, often in environments with limited resources and support [21–23]. The confluence of these challenges is potentially detrimental, increasing caregivers’ susceptibility to mental health issues. Ashrafizadeh et al. [7] highlight the diverse sources of stress experienced by caregivers, including the physical demands of caregiving, the emotional challenge of dealing with behavioral symptoms of ADRD, and the economic strain of shouldering medical costs. Brodaty and Donkin [8] further noted that in the absence of adequate support structures, these sources of stress are likely to coalesce, intensifying the risk of psychological distress and depression among caregivers.

### Gender differences

A significant proportion of the participants were females (82.2%). This highlights the traditional caregiving role that women often shoulder, particularly in LMICs [24]. Despite the gender skew, the study did not find significant disparities in Quality of Life, Psychological Distress, or depression between the genders. This possibly indicates that the act of caregiving, not gender, is the primary driver of the psychological challenges faced. That said, socioeconomic conditions affecting genders differently, such as females predominantly relying on relatives for income and males relying on business activities, might intersect with caregiving roles in subtle, yet crucial ways.

### Quality of life & age

Contrary to expectations, this study did not find a statistically significant association between age and Quality of Life (QoL). The results indicated that age, whether lower or higher, did not significantly impact QoL among the caregivers. This suggests that the challenges impacting caregivers' QoL may be more universally experienced across different age groups rather than being particularly pronounced in younger caregivers. While previous research has highlighted the dual stress faced by younger caregivers in balancing caregiving responsibilities with their personal and professional lives [4, 23, 25, 26], our findings suggest that this stress does not significantly differentiate QoL across age groups. This may indicate the need to consider factors other than age when addressing QoL among caregivers. Quality of Life & Age: Interestingly, this study found a lower age to be associated with a decrease in QoL. This implies that younger caregivers, possibly in their prime working years, face the dual stress of caregiving and managing their personal and professional lives. This aligns with the hypothesis that caregivers’ QoL is inversely correlated with distress. Furthermore, this finding aligns with existing literature. For instance, research has shown that younger caregivers often experience the dual stress of managing their caregiving responsibilities along with their personal and professional lives [4, 23, 25, 26]. This could be particularly challenging during their prime working years as career development and personal growth are crucial.

### Children and psychological distress

The finding that having children is linked to decreased psychological distress is intriguing and noteworthy in a caregiving context. Initially, one might assume that having children equates to increased support and reduced stress levels. However, the dual responsibility of caregiving for loved ones and parenting can be overwhelming, resulting in increased stress. Folkman and Lazarus [27] identified role conflicts as a source of stress, as individuals struggling to balance competing demands. Pearlin et al. [28] demonstrated that caregivers who take on multiple roles are susceptible to role strain, leading to feelings of inadequacy and heightened psychological distress. Given the complexity of caregiving and the need to attend to the needs of multiple children, caregivers of more children may be particularly vulnerable to this strain.

Our results suggest that having children is associated with a significantly lower risk of psychological distress among caregivers (Adjusted OR 0.09, 95% CI: 0.01–0.67, *p* = 0.019). This finding is crucial, as it highlights the protective effect of having children against psychological distress, in contrast to the potential increased burden of managing multiple roles. Additionally, having children was associated with better quality of life (QoL) for caregivers, possibly due to the support that children can provide, which can alleviate some of the caregiving burden [29]. Moreover, psychological distress is significantly associated with poorer QoL, highlighting the interconnectedness of these factors [19].

Interestingly, having children was also found to predict a lower risk of depression among caregivers. This nuanced finding suggests that, while the presence of children can provide significant emotional and practical support, the overall number of children might still influence the level of psychological distress experienced by caregivers. This complexity underscores the need for further research to explore the balance between caregiving demands and the support provided by children in different caregiving contexts.

### Strengths and limitations

This study offers a fresh perspective on a previously under-researched demographic. The use of purposive sampling and standardised scales with proven internal consistency underpins the strengths of this study. However, this methodology has some limitations. While purposive sampling can provide targeted insights, it does not necessarily imply rigor, particularly in quantitative studies, and may introduce a selection bias. The sample size of 90 participants, though justified by its depth and focus, may limit the generalisability of the findings and require further validation in larger samples. Additionally, the cross-sectional nature of this study prevented causal inferences. The focus on one district, although detailed, may not be representative of the entire Ugandan context.

### Implications for policy and practice

The results have profound implications for the development of healthcare policies and support services in Uganda and similar LMIC settings. There is a pressing need for structured caregiver support systems that incorporate mental health screenings, counselling services, and community-based respite care programs. Given the gendered nature of caregiving, gender-responsive interventions that recognize the unique challenges faced by male and female caregivers could be beneficial. While the study provides valuable insights into the experiences of caregivers in this district, further research incorporating larger and more diverse samples and longitudinal designs is needed to validate and expand these results. Policymakers and practitioners should use these findings as a foundation for more extensive studies that can inform broader and more generalisable policies and interventions.

## Conclusion

Amidst the challenges presented by Alzheimer's Disease and related dementias, caregivers bear a substantial emotional and psychological burden that is often overlooked. This study highlights how factors such as gender, age, and socioeconomic status intersect with caregiving in the Ugandan context, thereby providing a nuanced understanding of these challenges. These findings underscore the urgent need for comprehensive interventions at the community, healthcare, and policy levels. Addressing the psychological well-being of caregivers is not only an act of empathy, but also a crucial component in the holistic care of patients with ADRD. This research calls for targeted support systems to alleviate caregiver stress and enhance the quality of life of both caregivers and patients.

## Data Availability

The datasets used and analyzed during the current study are available from the corresponding author upon reasonable request.

## Abbreviations

ADRD: Alzheimer’s Disease and Related Dementias
QoL: Quality of Life
K10: Kessler Psychological Distress Scale
C-DMEQOL: Caregiver Dementia Quality of Life Measurement
CES-D: Center for Epidemiologic Studies Depression Scale
OR: Odds Ratio
CI: Confidence Interval
UBOS: Uganda Bureau of Statistics
VHTs: Village Health Teams
